# Design of a stable cell line producing a recombinant monoclonal anti-TNFα antibody based on a CHO cell line

**DOI:** 10.1186/s40064-016-3213-2

**Published:** 2016-09-15

**Authors:** E. V. Voronina, Y. A. Seregin, N. A. Litvinova, V. I. Shvets, R. R. Shukurov

**Affiliations:** 1 PHARMAPARK LLC, Bldg. 1, 8 Nauchny proezd, Moscow, Russian Federation; 2 M.V. Lomonosov Moscow State Academy of Fine Chemical Technology, Moscow, Russian Federation

**Keywords:** Monoclonal anti-TNFα antibody, Tumor necrosis factor alpha, CHO-DG44, Dihydrofolate reductase mediated gene amplification

## Abstract

Recombinant monoclonal antibodies (mAbs) against tumor necrosis factor alpha are widely used in the biopharmaceutical therapy of autoimmune diseases. Currently, a large number of drugs based on these antibodies are available. Accordingly, the development of these products for the Russian market is an important goal. The aim of the current study is to describe the development of one such technology. CHO-DG44-derived cell lines producing mAb were developed using two strategies, one based on individual clones and the other based on cell pools. To obtain recombinant cell lines with highly amplified genes of interest, the clones underwent dihydrofolate reductase-mediated gene amplification. Using the best strategy for the selection and amplification of mAb-producing clones, we achieved the production of more than 1 g/L in small scale, non-optimized conditions.

## Background

Genetically engineered biological agents designed from monoclonal antibodies (mAbs) have been a leading technology for pharmacotherapy in the last decade (Kalden 2002). The introduction of targeted therapy based on mAbs has promoted many important scientific achievements in rheumatology (Alonso-Ruiz et al. 2008). Specifically, recognition of the role of tumor necrosis factor alpha (TNF-α) in the pathogenesis of inflammatory rheumatic diseases has expanded the scope of drugs for these diseases (Alonso-Ruiz et al. 2008; Schiff et al. 2005).

Currently, adalimumab (otherwise known as Humira) is the representative TNF-α blocker for the treatment of rheumatoid arthritis (RA) and other common inflammatory arthropathies (Furst et al. 2007; Nasonov 2007). This drug is a completely human antibody that determines its reduced immunogenicity, which is manifested particularly in the reduction of allergic reactions (Furst et al. 2007; Nasonov 2007; Scott and Kingsley 2006; Wurm 2004; Chu and Robinson 2001). Worldwide, adalimumab has largely been successful in the treatment of inflammatory rheumatic diseases, including in multiple random clinical trials (Scott and Kingsley 2006). In the USA, adalimumab has been approved for the treatment of RA, psoriatic arthritis, ankylosing spondylitis, Crohn’s disease, ulcerative colitis, and chronic plaque psoriasis, but in Russia, this drug has only been registered for the treatment of RA. In addition, the annual sales volume of Humira in the USA is about $2.6 billion (Scott and Kingsley 2006), highlighting its importance as a commonly prescribed drug.

Part of a strategy to develop the pharmaceutical industry in Russia includes reducing the dependence of the Russian market on imported drugs and improving the availability of new drugs. The development of modern high-technology production of domestic drugs based on mAbs is a challenge for the Russian medical community. Therefore, the aim of this study was to obtain a stable cell line producing a mAb for a TNF-α blocker and to characterize the purified antibody in comparison with the drug Humira (AbbVie, USA).

This paper is describing the development of a cell line based on Chinese Hamster (Cricetulus griseus) Ovary (CHO) cells. Among mammalian cell lines used in biopharmaceutical production, CHO cells and their derivatives are the most ubiquitous (Urlaub and Chasin 1980; Derouazi et al. 2006).

For the production of therapeutic proteins, the most widely used mammalian expression system in industrial production is gene amplification using dihydrofolate reductase-deficient (DHFR−) CHO cells with DHFR-mediated gene amplification. Methotrexate (MTX) binds to and inhibits the DHFR enzyme, leading to cell death. However, DHFR− CHO cells, being transfected with an expression vector containing the DHFR gene, can develop resistance to MTX. Because the amplification unit consists a specific gene of interest, either co-linked to DHFR in the same expression vector or residing adjacently in the host chromosome, is co-amplified (Cacciatore et al. 2010).

Because single-step, high-level resistance to MTX may result in cells synthesizing an MTX-resistant DHFR mutant, or in cells with altered MTX-transport properties, gene amplification is usually achieved by selection for resistance to gradually increasing concentrations of MTX in multiple steps (Cacciatore et al. 2010; Chusainow et al. 2009). However, clonal variation in foreign protein expression is significant because clones can still acquire MTX resistance by mechanisms other than DHFR-mediated gene amplification, despite stepwise selection. Therefore, a tedious and labor-intensive effort for DHFR-mediated gene amplification is required to obtain recombinant CHO (rCHO) cell clones with a high expression level of the target gene. To do this, two strategies, one based on individual clones and the other on parental cell pools, are commonly used. In individual clone-based selection, individual parental clones, usually isolated by the limiting dilution method in 96-well culture plates, are independently grown in increasing concentrations of MTX. Although the final clones that are resistant to high levels of MTX are derived clonally, they become heterogeneous with respect to the expression of the foreign protein (Cacciatore et al. 2010; Chusainow et al. 2009).

Several CHO-derived sublines differ in the activation status of the dihydrofolate reductase (DHFR) gene, which through the inhibition of DHFR, allows the amplification of foreign genes on genetic constructs that are introduced into the cells. In particular, the subline CHO-DG44 was developed using chemical mutagenesis. This cell line contains no active DHFR alleles (Lai et al. 2013) and is suitable for generation of stable cell lines producing recombinant proteins (Kim et al. 2001; Seung and Min 2005).

In this study two subcloning strategies were evaluated to produce high levels of the protein of interest in CHO-DG44 cell line, one based on individual clones and the other based on cell pools.

## Experimental section

### Plasmid construction

The plasmids pOptiVecTOPO and pcDNA3.3TOPO (Invitrogen, USA) were used as expression vectors. The codon-optimized synthetic gene cDNA sequences of the heavy chain (HC) and light chain (LC) mAb of Adalimumab (pUC57 HC-Adalimumab and pUC57 LC-Adalimumab, respectively) for CHO cells were ordered from Service-gene (St. Petersburg, Russia). The plasmids were modified by transferring DNA fragments encoding the HC and LC genes from the donor vectors to the plasmids. To do this, the plasmids were treated with the restriction enzymes BamHI, XbaI, and AgeI (Fermentas, Lithuania). Separation of the DNA fragments was performed by electrophoresis in a 1.0 % agarose gel using the Sub-Cell GT System (Bio-Rad, USA). For elution of DNA fragments from the gel, we used the QIAquick spin column kit (Qiagen, USA). Then, the generated fragments were ligated together with T4 DNA ligase (Thermo Fisher Scientific, Lithuania) to create the following expression vectors: pcDNA-LC Adalimumab; pOptiVec-HC Adalimumab; pOptiVec-LC Adalimumab; and pcDNA3.3-HC Adalimumab.

To obtain the desired concentration of DNA for the transient transfection of CHO cells, we transformed competent XL1 *E. coli* cells by electroporation with the obtained ligation mixture. The contents of the isolated plasmid DNA from the resulting bacterial clones were confirmed by restriction analysis.

For transfection of the highly pure (“transfection grade”) isolated plasmid DNA, we used the Plasmid Maxi kit (QIAGEN, USA). Proper assembly of the expression vector was verified by restriction analysis. The nucleotide sequences of both the genes and the adjoining regions were verified by sequencing.

### Culturing the CHO-S and CHO-DG44 cell lines

Cell cultures were carried out in 125 mL Erlenmeyer flasks in a CO_2_ Multitron Cell shaker-incubator (Infors HT, Switzerland) operating at a speed of 125 rpm in an atmosphere of 5 % CO_2_, at a temperature of 37 °C and 95 % humidity. Reseeding was performed every 3–4 days to a density of 0.3–0.5 × 10^6^ cells/mL. We used CD DG-44 (Life technologies, USA) and PowerCHO 2CD (Lonza, Switzerland) serum-free media supplemented with 8 mM L-glutamin. Cell counts and viability analysis were performed after staining with trypan blue (Panreac, Spain) using an automatic cell counter TC10 (Bio-Rad, USA).

### Transfection of CHO-DG44 and CHO-S cell lines

Transfection was performed using the following combination of expression vectors: pcDNA3.3 LC Adalimumab + pOptiVec HC Adalimumab and pOptiVec LC Adalimumab + pcDNA3.3 HC Adalimumab, using the lipophilic agent FreeStyle MAX (Invitrogen, USA).

One day prior to transfection, the cells were re-plated to a density of 0.5–0.6 × 10^6^ cells/mL. On the day of transfection, cell density was determined, and the cells were pelleted by centrifugation at 200*g* for 10 min at room temperature in an Allegra 25-R centrifuge (Beckman, Germany). The supernatant was removed by decantation, and the cells were suspended in FreeStyle™ CHO Expression Medium, containing 8 mM alanyl-glutamine (both reagents were from Invitrogen, USA) to a final density of 1.2–1.5 × 10^6^ cells/mL. Further transfection was performed in 6-well plates, according to the manufacturer’s instructions (FreeStyle CHO-DG44 Cells, Invitrogen, USA). Transfection efficiency was assessed by fluorescent microscopy of cells with the pEYFP plasmid and a blue color filter. The transfection efficiency was evaluated visually using a CKX41 microscope (Olympus, Japan).

**Fig. 1:**
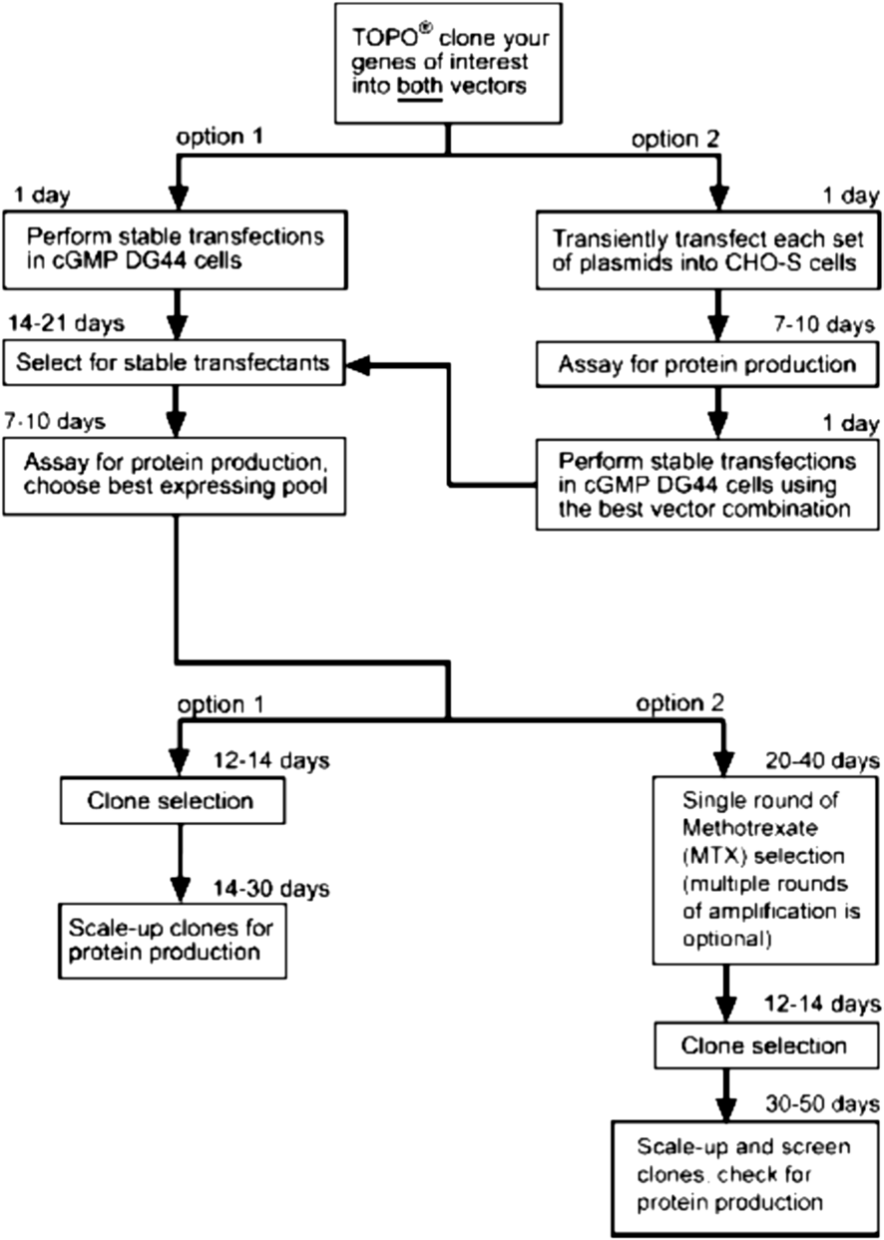
Clone selection scheme (adapted from User guide for Freedom™ DG44 Kit) and development of stable cell lines for protein production

According to the manufacturer’s instructions, subsequent selection of the transfected clones was performed as shown in the schematic representation below (Fig. 1).

### Selection of individual clones

**Fig. 2:**
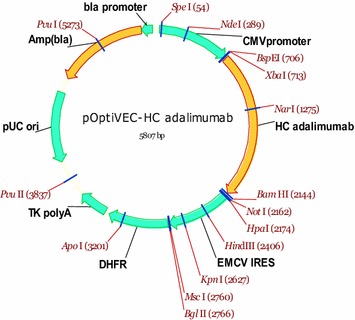
Construct of pOptiVEC-HC adalimumab, containing the sequence of adalimumab heavy chain. HC adalimumab, synthetic gene of heavy chain mAb for adalimumab, codon optimized sequence; CMV promoter, early gene promoter of human cytomegalovirus HCMV IE1; EMCV IRES, IRES element from the encephalomyocarditis virus (EMCV); *DHFR*, dihydrofolate reductase

Limiting dilutions were used to select individual clones. After transfection for 24 h, the cells were suspended in CD OptiCHO Medium (Life technologies, USA) containing 500 μg/mL solution of G418 (Lonza, Switzerland) and 10 nM MTX at a density of 10,000, 5000 or 1000 cells/well. Hundred microliter of the cell suspension was added to every well of a 96-well plate, using 30–40 plates for each dilution. The plates were cultured in a CO_2_ incubator at 5 % CO_2_ at 37 °C and 95 % humidity for 14–20 days. After 12 days, the growth of cells in the wells was controlled under a microscope, registering the wells that were experiencing cell growth and division. Upon reaching 80–100 % confluence, the individual mini-pools were transferred into 24-well plates. After 5 days, the samples were analyzed for the expression level of the target antibody using the IgG-ELISA-BEST kit (Vector-Best, Russia). The selected pools with the highest productivity were subcultured into 6-well plates, and then the positive pools were re-selected for cell density and productivity by ELISA. Then the leading clones were transferred into T-75 flasks, and further into 125 mL Erlenmeyer flasks in two media in parallel: CD OptiCHO Medium (Life technologies, USA) and ActiCHO SM (PAA, Austria) supplemented with 8 mM alanyl-glutamine, 25 nM MTX and 500 μg/mL G418. At every step the number of clones was reduced, basing on growth of cells, viability, and productivity (Fig. 2).

**Fig. 3:**
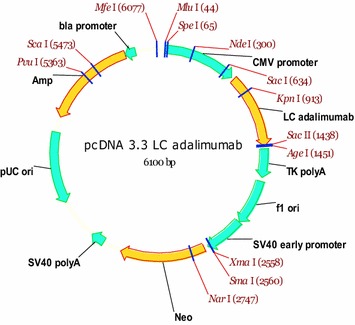
Construct of pcDNA-LC adalimumab, containing the adalimumab light chain. LC adalimumab, the synthetic gene of the light chain mAb for adalimumab, codon optimized sequence; CMV promoter, the early gene promoter of human cytomegalovirus HCMV IE1; *Neo* neomycin phosphotransferase

For the further selection of the clones, three rounds of amplification were carried out in mini-pools at three concentrations of MTX: 25, 500 and 1000 nM. Series of batch cultures were performed for 10 amplified pools with subsequent evaluation of IgG concentration by ELISA. Then, the mini-pools were subcloned using limiting dilution in 96-well plates as described above. From the 192 clones evaluated in 24 well plates, 60 clones were chosen based on the ELISA data with further reduction to 13 clones. Further batch cultures were carried out in 125 mL shaker flasks with subsequent evaluation of growth characteristics and productivity by ELISA, affinity chromatography, and electrophoresis (polyacrylamide gel electrophoresis under reducing conditions followed by Coomassie staining). Based on the results of total productivity, growth characteristics, and stability, subclone 30 was selected for the further analysis (Fig. 3).

### Alternative protocol: amplification of the total pool of transiently transfected cells

One day after transfection the cells were transferred to a selection medium, OptiCHO, containing 500 nM MTX and 500 μg/mL G418. Every 2–3 days, the medium was changed by centrifugation at 200×*g* for 5 min and resuspension in the fresh medium. After 40 days, when the viability was over 90 %, the pool was transferred into a medium containing an increased concentration of MTX (1000 nM). The amplification process was performed in a similar manner as described above. The last stages of amplification of the common pool were carried out at a concentration of 2.5 μM and 5 μM MTX, correspondingly. Upon reaching a cell viability of greater than 90 %, the total pool was subjected to limiting dilutions to select individual clones. From the initial 40 subclones, 20 subclones were selected on the basis of their specific productivity and overall productivity in the production batch. The resulting subclones were evaluated for expression stability with or without the MTX selective agent during 60 generations. For the best clones, production batches were carried out in the different media. Based on the total productivity, growth characteristics, and stability, subclone 26 was selected for further investigation.

### Colorimetric assay for tumor necrosis factor using WEHI 164 cells

Neutralizing activities of adalimumab against human TNF-α were measured on the mouse WEHI 164 cell line treated with actinomycin D according to the method described previously (Austgulen et al. 1986; Khabar et al. 1995). Briefly, WHEI 164 cells were seeded in triplicate at 1 × 10^4^ cells/well into a 96-well plate and cultured in RPMI 1640 medium supplemented with 10 % (v/v) FBS for 20 h. Then, serially diluted antibodies (final concentration: 0.5–50 ng/mL) in the medium containing 2 µg/mL actinomycin D were added to the cell culture together with 0.1 ng/mL of human TNF-α. The cells were incubated for an additional 20 h at 37 °C and cell viability was analyzed using a colorimetric MTT-based Cell Growth Determination kit (Sigma, St. Louis, MO). The ED50 value was calculated by complex sigmoid non-linear regression analysis using Sigma plot software (Systat software, Inc. Richmond, CA).

### Determination of the concentration of monoclonal anti-TNFα antibody

Concentration of anti-TNF-α mAb was measured by ELISA using IgG-ELISA-BEST kit (Vector-Best, Russia), calibrated for Humira (AbbVie, USA). SDS-PAGE electrophoresis under reducing conditions was performed according to the standard procedure in a 12 % Laemmli gel followed by staining with Coomassie-R250 to check the ratio of heavy and light chains in the supernatant.

## Results and discussion

At the initial stage, genes encoding heavy (HC) and light (LC) chains of Adalimumab monoclonal antibody (mAb) with optimized codon composition were synthesized. Gene sequences were obtained from publicly available sources (DrugBank and patent databases). To confirm the sequence of monoclonal anti-TNFα antibody, a commercial drug, Humira, was subjected to peptide mapping using mass spectrometry-grade endoproteinases Chymotrypsin, Asp-N, Glu-C (Thermo Scientific, USA).

For extracellular secretion of the antibody into the culture supernatant, the sequences of the LC and HC were linked to signal peptides taken from human albumin gene.

It has been shown that various genetic engineering approaches may be used for creating genetic constructs expressing mABs in CHO cells (Steven et al. 2013; Ng et al. 2010). In this paper, were used a traditional approach based on the pOptiVecTOPO and pcDNA3.3TOPO plasmid vectors as carriers of the individual genes of the HC and LC antibody chains.

It is known that one disadvantage of this approach is the possible survival of non-expressing clones during selection (Steven et al. 2013; Bardor et al. 2012; Schlatter et al. 2005). Another drawback is the lack of control over the ratio of expression of HC and LC. The LC is required to facilitate the folding and release of HC from binding immunoglobulin protein to form a complete IgG monomer. Each gene is under the control of its own promoter and is transcribed separately. According to the literature, the expression of the LC in excess has a positive effect on the quality of the mAbs, and the ratio of LC:HC expression can affect mAb qualities such as glycosylation and aggregation (Schlatter et al. 2005; Lee et al. 2009). On the other hand, an excess of HC can cause ER stress and proteasome overloading, creating a burden on the cell machinery that can inhibit cell proliferation (Lee et al. 2009).

Thus, CHO-DG44-derived cell lines producing mAb were developed using two strategies, one based on individual clones and the other based on cell pools. To obtain recombinant cell lines with highly amplified genes of interest, the clones underwent dihydrofolate reductase (DHFR)-mediated gene amplification.

According to the first strategy, individual clones were selected by limiting dilutions, followed by amplification of each separate clone.

At the first step, the combination of selected genetic constructs was transfected into CHO-DG44 cells. Twenty-four hours after transfection, selection of cells was performed. After 14–20 days of culture in 96-well plates, cell growth was analyzed using light microscopy. Growth of cell colonies was found in 10 % of the wells at a dilution of 100 cells/well (1000 cells/mL), in 25 % of the wells at a dilution of 500 cells/well (5000 cells/mL), and in 60 % of the wells at a dilution of 1000 cells/well (10,000 cells/mL). At this stage, 310 clones were selected for further analysis.

After an initial screening of the clones in 96-well plates using ELISA, were selected 68 clones that had the highest expression level.

**Fig. 4:**
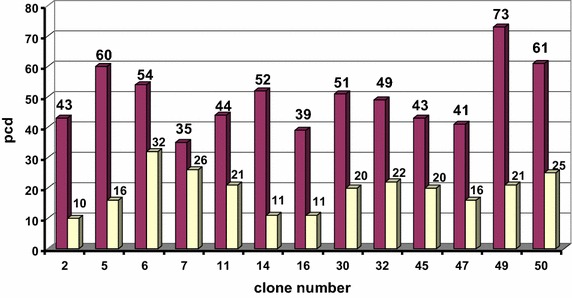
Stability of the antibody production for the best subclones selected from the individual parental clones. Specific productivity (pcd) was indicated before and after continuous cultivation for 60 generation

For a more precise analysis of the productivity for the best growth characteristics, the clones were transferred to 24-well plates. Then, the leading 40 clones were cultured in T75 flasks and transferred into 125 mL flasks. For each clone, growth characteristics were controlled and productivity was measured after 4 days of batch cultivation. For 10 the most productive mini-pools, 3 rounds of amplification were performed, at 25, 500 and 1000 nM MTX. After amplification, subcloning was carried out with three prospective mini-pools using limiting dilutions. As a result, 13 clones were selected with a specific productivity of 9–73 pcd [pg cell (−1) day (−1)] (Barnes et al. 2007) (Fig. 4). Specific productivity was calculated using the following formula:

$$ pcd = \frac{{p_{n} - p_{0} }}{n} \cdot \frac{{ln\left( {\frac{{CD_{n} }}{{CD_{0} }}} \right)}}{{CD_{n} - CD_{0} }}, $$

where P_0_ and P_n_ are the concentrations of the antibody in the supernatant at time zero and the last day of cultivation, respectively, CD_0_ and CD_n_—cell densities on the corresponding days and n—culture duration in days.

In total 13 leading clones were selected from 3 positive mini-pools. They were undergone an investigation of the production stability in continuous cultivation for 2 months. The drop in productivity was found to be 30–70 % during this time (Fig. 4). According to the stability and growth characteristics, we selected clone #30 for further analysis. When this clone was cultured for 8 days in fed-batch, the maximum cell density was 18 × 10^6^ cells/mL and productivity was more than 1 g/L.

All clones developed using this strategy was found to have propensity to aggregate. As known, for batch suspension cultures, cell aggregation hinders accurate cell counting, monitoring and control of the cellular environment. Transport of nutrients to, and products from, the cells may be impaired, cluster formation dramatically influences the growth behavior of the cells, cells within aggregates show a strongly reduced specific proliferation rate, apart from, shear forces exerted on large aggregates cause a considerably higher specific death rate than those exerted on single cells, reducing the specific growth rate up to 50 %. This issue was resolved using dextran sulfate based anti-clumping agent (Lonza, Switzerland). But when the protein was subjected to purification on MabSelect column (GE Healthcare, USA), significant protein aggregation and loss was observed. Moreover, all clones grew slowly (doubling time 40–50 h), and the best one, #30, reached the maximum cell density at 12.3 × 10^6^ cells/mL. As a result, fed-batch in shaker flasks gave the effective productivity of only 0.4 g/L (this value indicates the yield after the first purification step).

According to the second strategy, initially we subjected a pool of transiently transfected cells to amplification. Transfection was performed in a similar manner as described above. The cells were cultured in a selective medium for 40 days, initially containing 25 nM MTX and further increased to 5000 nM. When cell viability reached more than 90 % for the generally amplified pool, we evaluated productivity in a fed-batch.

**Fig. 5:**
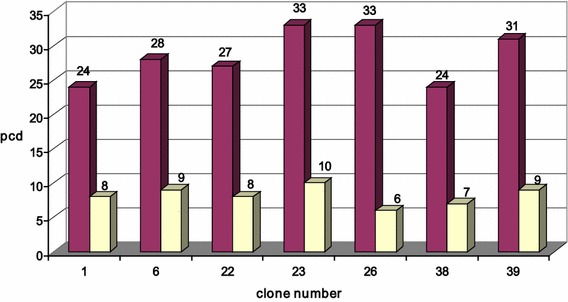
Stability test for antibody pcd of best subclones selected on the basis amplification of the total pool of transiently transfected cells after 4 days of cultivation during 60 generation

Amplified pool was subcloned using limiting dilutions as described above. From 40 subclones, 20 leading ones were selected and assessed in terms of overall productivity. The best 7 subclones were evaluated for stability with or without selective agent during 60 generations. Productivity per cell using the second selection strategy was significantly lower (Fig. 5). On the other hand, all clones generated using the second strategy performed much better in terms of cell growth. They didn’t aggregate, their doubling was typical for CHO cells (23–35 h). For the leading clone, #26, maximum cell density in a fed-batch process was 62.7 × 10^6^ cells/mL, and process duration was 14 days. Moreover, no product aggregation was detected at purification. Consequently, the effective productivity after the first purification step reached 1.3 g/L. Certainly, such data as 1–1.3 g/L is a little low, however these are non-optimised conditions, wherein for one of the producer has been shown a high productivity and stability of expression of prolonged cultivation. Further work is needed to optimize the culture conditions for a further increase in productivity of clone-producing.

It’s important to note, the proteins obtained by both methods is identical in respect to its capacity to neutralize TNF-activity indeed. We carried out an in vitro assay to compare the binding capacity to soluble recombinant human (rh)TNFα and in vitro study to compare the inhibition the binding of rhTNFα to TNFα receptors. These assays showed the protein has similar activity for both cases.

Thus, despite the lower specific productivity of the clones generated according to the second strategy, they were able to reach much higher cell density that caused better overall productivity.

Further investigation is needed to understand the issues associated with the clones developed using the first strategy. Current data suggest that intensive amplification caused an excess of heavy chain that leaded to overload of translational machinery, insufficient folding and ER stress. In this case it seems unlikely to find any culture conditions to make these cell lines working without aggregation issues. On the other hand, the second strategy was not as effective to attain high HC expression, LC was in excess, and no stress emerged.

This hypothesis was indirectly confirmed by measuring HC/LC ratio in the supernatants by SDS-PAGE. All clones from the second selection produce an excess of LC, while the clones from the first selection had almost 1:1 ratio (data not shown).

## Conclusions

Two independent strategies were evaluated for selection and amplification of antibody-producing clones originated from CHO-DG44 cell line. Completely different parameters were reached for the resulting clones, both in terms of productivity and culture characteristics. The best strategy implemented in our study generated the cloned producing more than 1 g/L of the target antibody in shaker flasks in non-optimized conditions.

The results indicate that maximum specific productivity per cell should not be the only goal of cell line development. Intensive amplification may cause undesirable effects on cell growth and protein quality that countervail the benefits of high pcd. Thus, rational balance should be kept between specific productivity and maintained phenotype.
